# Circulating Cell Free Tumor DNA Detection as a Routine Tool for Lung Cancer Patient Management

**DOI:** 10.3390/ijms18020264

**Published:** 2017-01-29

**Authors:** Julie A. Vendrell, Frédéric Tran Mau-Them, Benoît Béganton, Sylvain Godreuil, Peter Coopman, Jérôme Solassol

**Affiliations:** 1 CHU Montpellier, Arnaud de Villeneuve Hospital, Department of Pathology, 34295 Montpellier, France; J-vendrell@chu-montpellier.fr (J.A.V.); f-tran_mau_them@chu-montpellier.fr (F.T.M.-T.); 2 IRCM, Institut de Recherche en Cancérologie de Montpellier, 34298 Montpellier, France; benoit.beganton@inserm.fr (B.B.); peter.coopman@inserm.fr (P.C.); 3 INSERM, U1194, 34298 Montpellier, France; 4 ICM, Institut Régional du Cancer de Montpellier, 34298 Montpellier, France; 5 Université de Montpellier, 34000 Montpellier, France; 6 CHU Montpellier, Arnaud de Villeneuve Hospital, Department of Microbiology, 34295 Montpellier, France; S-godreuil@chu-montpellier.fr

**Keywords:** circulating DNA, molecular diagnosis, targeted therapies, routine practice, lung cancer

## Abstract

Circulating tumoral DNA (ctDNA), commonly named “liquid biopsy”, has emerged as a new promising noninvasive tool to detect biomarker in several cancers including lung cancer. Applications involving molecular analysis of ctDNA in lung cancer have increased and encompass diagnosis, response to treatment, acquired resistance and prognosis prediction, while bypassing the problem of tumor heterogeneity. ctDNA may then help perform dynamic genetic surveillance in the era of precision medicine through indirect tumoral genomic information determination. The aims of this review were to examine the recent technical developments that allowed the detection of genetic alterations of ctDNA in lung cancer. Furthermore, we explored clinical applications in patients with lung cancer including treatment efficiency monitoring, acquired therapy resistance mechanisms and prognosis value.

## 1. Introduction

Lung cancer is the most common cause of cancer death around the world. About 80%–85% of lung cancer cases are non-small-cell lung cancer (NSCLC) patients, the remaining 15%–20% are small-cell lung cancer (SCLC) [1]. NSCLC is divided into three categories called: adenocarcinoma, squamous-cell adenocarcinoma and large cell adenocarcinoma. Among them, adenocarcinoma cases account for around 40% of NSCLC patients. The prognosis for NSCLC is low with a five-year survival rate of less than 20%, and is even worse for SCLC with a five-year survival rate of less than 5% [1].

For a long time, the first-line treatments have been surgery, chemotherapy or radiotherapy. However, the discovery of several oncogenic driver mutations in patients with NSCLC, adenocarcinoma cases in particular, has allowed the development of personalized treatments based on these specific molecular alterations. Therefore, *EGFR* (epidermal growth factor receptor) mutations account for up to 15% of adenocarcinoma and primarily occurred in the tyrosine kinase (TK) domain of the gene. More than 80% of these mutations consist of in-frame deletions in exon 19 and the L858R point mutation in exon 21. Such mutations induced a constitutive activation of EGFR, making it a potential therapeutic target. Thus, *EGFR*-mutated patients can benefit from a specific first-line treatment specifically the TK inhibitors (TKI) that competitively inhibits fixation of adenosine triphosphate (ATP) in the catalytic binding site of TK domain. Other driver biomarkers in lung cancer (point mutations, rearrangements or amplifications in specific genes including *KRAS*, *NRAS*, *HER2*, *BRAF*, *ALK*, *RET*, and *ROS1*) have also been proposed and some of them might provide additional information for clinical decision-making.

Unfortunately, side effects of personalized treatments have emerged. Among them, the appearance of the T790M mutation located in exon 20 of *EGFR* systematically results in cancer relapse, generally within 1–2 years. The T790M mutation is present in about half of the lung cancer patients with acquired resistance, and is reported to increase the affinity of the receptor to ATP, relative to its affinity to TKIs [2]. Identification of such mutations is required to propose second-line treatment. Recently, third-generation EGFR inhibitors, such as osimertinib, mereletinib or rociletinib, have been proposed as relevant therapeutics that could specifically disrupt the growth of *EGFR* T790M-positive tumors and thus increase patient survival [3,4,5].

## 2. Tumor Tissue Biopsy Limitations

Molecular characterization of tumors became mandatory, not only for patients to receive the right treatment, but also to follow the evolution of the molecular characteristics and, accordingly, to adapt treatments [6]. Tissue biopsies remain the gold standard to assess molecular alterations. However, this strategy presents several limitations that can impair patient treatment. Indeed, access to tumor tissues is not always optimal. Many patients with NSCLC are diagnosed at an advanced stage of the disease that makes the surgery or the biopsy difficult and even sometimes dangerous. Thus, complications from intrathoracic biopsies have been reported in 17.1% cases in a series of 211 biopsies [7]. In addition, the quality/quantity of the available tumoral material and *EGFR* genotyping failed in approximately 5% of the cases [8]. Finally, the intratumoral heterogeneity of *EGFR* mutation status has been described in several studies (ranges from 13.9% to 27%; [9]) demonstrating that tumor biopsy do not systematically reveal the complete genomic landscape of the whole patient tumoral cell population. Altogether, these issues related to tissue biopsy analysis failure resulted in an unknown *EGFR* status and excluded some patients that could have been eligible to TKI treatment.

**Figure 1:**
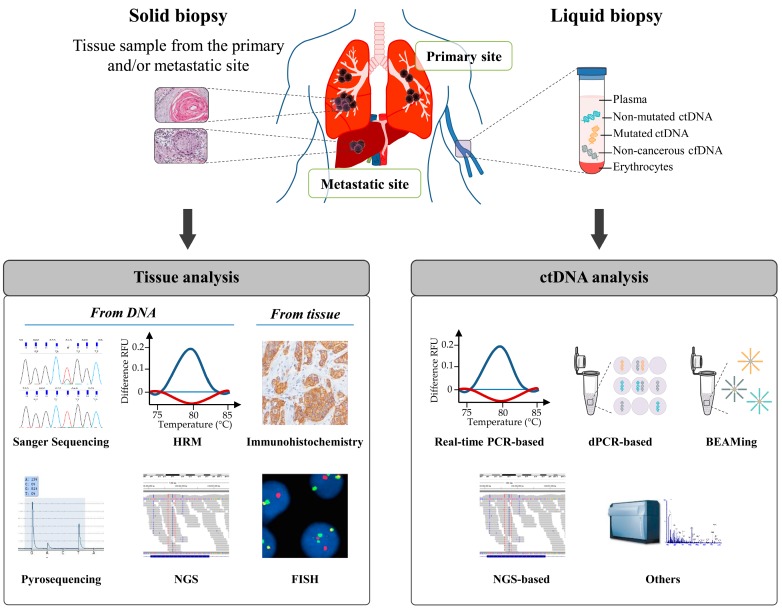
Overview of the available techniques to detect alterations from solid or liquid biopsies. The left side describes the conventional techniques that use tissue sample as starting material, specifically Sanger sequencing, pyrosequencing, High Resolution Melting (HRM), Next-Generation Sequencing (NGS) and Immunohistochemistry. The right side highlights the different methods available for aberration detections from liquid biopsy. They include, in particular, real-time polymerase chain reaction (PCR)-based methods, digital PCR (dPCR), Beads, Emulsion, Amplification, and Magnetics (BEAMing) and NGS-based methods. DNA strand in blue corresponds to non-mutated circulating tumoral DNA (ctDNA), in orange to mutated ctDNA and in grey to non-cancerous cell-free DNA (cfDNA). For each technique, a representation of the principle or the result is given as illustration.

Given these limitations, exploring alternative practical, economical and less invasive techniques to monitor the EGFR TKI therapy in NSCLC is absolutely needed. Noninvasive approaches, based on samples of plasma or serum, have shown great potential in monitoring the EGFR TKI therapy in recent years. Among the different materials derived from liquid biopsies, ctDNA has been successfully applied to detect *EGFR* mutations in NSCLC patients and can give similar molecular information as those given by invasive tumor biopsies [10] (Figure 1). In addition, the dynamic changes in ctDNA *EGFR* mutation status may predict clinical outcome of EGFR TKI therapy [11]. In patient drug resistance instances, one alternative to improve early detection rate and overcome the limitation of repeated tissue sampling is to perform genomic analysis using other liquid biopsy markers such as circulating tumor cells (CTCs), circulating RNA, circulating miRNA, platelet markers, etc. Since the use of these different markers for lung cancer management has previously been reported, it will not be discussed here [11,12,13,14,15,16,17].

Hereby, we summarized different technical approaches available that have been proposed for the detection of molecular events from ctDNA and considered their possible applications in hospitals and routine laboratories for the management and monitoring of patients with lung cancer.

## 3. The Biology of cfDNA and Circulating Tumoral DNA (ctDNA)

New opportunities arose with the discovery of circulating cell-free DNA (cfDNA) in unaffected individuals [18]. Application includes different fields specifically the non-invasive prenatal diagnosis with the use of cell-free fetal DNA (cffDNA; [19]) and cancer with the use of circulating tumor DNA (ctDNA; [20]).

Origin and mechanisms of cfDNA release in bloodstream are still not completely documented. It is however widely accepted that several conditions such as inflammation, heavy smoking, or pregnancy can induce cfDNA release from cells into the systemic circulation [21,22,23]. As for patients suffering from heart injury, cfDNA increase over the first 48 h in emergency intensive care unit predicts fatal outcome [24]. The source of ctDNA is also likely multiple and mainly included cell lysis induced by apoptosis and/or necrosis of primary tumors and metastases [25,26] (Figure 1).

cfDNA and ctDNA are highly fragmented with a median size of 170 base pairs or less, which corresponds to the DNA wrapped around a nucleosome plus a linker fragment [27,28]. Several studies have tried to clarify the alleged mechanism of ctDNA (necrosis versus apoptosis) depending on the size of the ctDNA, however, results remain controverted [26,29]. Indeed, Wang et al. [30] and Gao et al. [31] reported that ctDNA is longer than normal cfDNA [30,31]. Paradoxically, Diel et al. [27] and Moulière et al. [29] observed a lower size of ctDNA. Most importantly, ctDNA is probably composed of short and long fragments with genetic aberrations specifically carried by the shorter ones. This hypothesis has been recently validated in hepathocellular carcinoma patients [32] and in lung cancer patients [22].

## 4. Technical Approaches for ctDNA Detection and Analysis

Preanalytical conditions may certainly play a crucial role in ctDNA detection. Due to different aspects of ctDNA (high fragmentation, contamination by non-tumoral cfDNA, low amounts and clearance), detection of molecular events from ctDNA materials remains a challenge and requires adapted and ultrasensitive analytical assays. Therefore, specific formaldehyde-free cfDNA collection tubes have recently been commercialized. Such processes not only stabilize but also prevent the release of genomic DNA from nucleated blood cells and reduce the need of immediate plasma preparation. In addition, these tubes allow transport and storage at room temperature and are highly adapted to hospital shipment procedures.

Comparative analysis of ctDNA in plasma and serum have shown that plasma represents the best tool to monitor NSCLC patients in clinical practice [33]. However, ctDNA dilution in patient’s cfDNA highly limits liquid biopsy’s detection of genetic alteration. Only a few thousands of copy number of cfDNA per milliliter of plasma could be extracted, among which only a small fraction is clinically relevant. Therefore, since genetic alterations that need to be detected from ctDNA are diluted by both the non-tumoral cfDNA and by the non-mutated ctDNA, highly sensitive and specific detection methods are required to provide a relevant ctDNA-based diagnosis. This concern has led to the improvement and the development of several methods of detection such as real-time polymerase chain reaction (PCR), digital PCR (dPCR), Next-Generation Sequencing (NGS), Beads, Emulsion, Amplification, and Magnetics (BEAMing) (Table 1). These methods can be classified into two groups: (i) the targeted approaches that allow detection of specific alterations; and (ii) the untargeted approaches that allow identification of events without a priori, in particular whole-exome sequencing or whole-genome sequencing.

### 4.1. Real-Time PCR-Based Methods

Allele-specific amplification combined with real-time PCR are commonly used in clinical setting to detect mutations from formalin-fixed paraffin-embedded (FFPE) tumor tissues. Even commercial kits based on the same principle have been developed and are widely used to detect single nucleotide variation (SNV) or small insertion/deletion (indels) (therascreen kit from Qiagen, Hilden, Germany and cobas^®^ from Roche Diagnostics, Meylan, France). However, as they were not fully adapted to the detection of rare genetic events, specific and more sensitive PCR-based methods have been engineered. Notably, custom-designed coamplification at lower denaturation temperature (COLD-PCR) [53,54] or Peptide Nuclei Acid-Locked Nucleic Acid (PNA-LNA) PCR clamp method [35,55,56] have been successfully applied to lung cancer samples. Briefly, COLD-PCR allows the enrichment of low-abundance mutations from a mixture of wild-type, regardless of whether they are known or unknown mutations. Therefore, lower denaturation temperature used during the PCR helps the amplification of heteroduplex mutant/wild-type sequence [34,57]. This PCR method has been further coupled with HRM, pyrosequencing, or Sequencing analysis of the harbored mutations identification [34].

PNA-LNA PCR clamp protocol takes advantage of the increased stability of PNA and LNA probes to highly bind DNA sequences compared to DNA duplex. In this approach, PNA probes firmly bind to DNA to specifically inhibit the amplification of the wild-type allele and thus, increase the specific detection of the mutant allele in real-time PCR cycling. An improved PNA-LNA PCR clamp method has been used to detect *EGFR* mutations in plasma samples [56].

Efforts were also focused on the improvement of allele-specific amplification technique. Indeed, probe-blocking methods have been engineered to block amplification of wild-type templates and thus, to increase detection sensitivity of mutant alleles. Therefore, minor groove binder (MGB) blocker oligonucleotide [37] and modified non-extendable primer blocker (NEPB) [36] have been developed and demonstrated the detection of mutation present at 0.1% in a background of wild-type DNA. Scorpion probes, for which higher sensitivity compared to Taqman probes has been demonstrated, also enable the detection of rare mutations [58,59,60,61,62].

Finally, as there is a tremendous and increased market for the detection of mutation from plasma specimens, new versions of commercial kits have been refined. In particular, the cobas^®^ *EGFR* Mutation Test v2 has been the first liquid biopsy test to be approved by the Food and Drug Administration (FDA) for the detection of *EGFR* mutations.

### 4.2. Digital PCR (dPCR)

dPCR relies on a real-time PCR, except that DNA templates are partitioned to obtain individual DNA molecule per entities (well, droplet or chamber) that are subsequently amplified by PCR and independently analyzed. Based on the Poisson distribution, it is assumed that small volume reaction compartments must contain 0 or 1 DNA molecules. After end-point PCR quantification of positive compartments, absolute concentration of the target is determined. Several digital PCR platforms are available and based on different process: microfluidic-chamber-based, micro-well chip-based and droplet-based [63]. The most common platforms in clinical laboratories are digital droplet PCR (ddPCR) in which samples are dispersed into thousands of droplets. Droplets containing mutated or non-mutated DNA strand can be discriminated by flow cytometry using fluorescent TaqMan-based probes [63], which allows sensitive detection of mutated ctDNA in a vast background of cfDNA.

Besides high sensitivity estimated at 0.01% to 0.1% [38], dPCR also has a relatively easy workflow, which can be implemented in a clinical setting [64]. Moreover, it has also been applied to detection of copy number variations (CNVs) in the blood sample of lung cancer patients [65]. One disadvantage is that dPCR only screens for known mutations, even if recent works demonstrated the feasibility of multiplex dPCR to detect *EGFR* and *KRAS* mutation in blood samples of cancer patients [40,66].

### 4.3. Beads, Emulsion, Amplification and Magnetics (BEAMing)

BEAMing is also a targeted approach based on the same principle as the emulsion PCR. Briefly, a first conventional PCR step is performed using primers specific of the targeted sequence that contain known tag sequences. Emulsion PCR of the amplicons is done in presence of tag-coupled magnetic beads that is easily purified. After single-base primer extension or hybridization with fluorescent mutant-specific probes, flow cytometric analysis allows the detection and quantification of mutant versus wild-type alleles [42]. In lung cancer samples, this technique already demonstrated its potency in the detection of *EGFR* activating mutations and the T790M resistance mutation from plasma DNA samples [41,67,68]. Like dPCR methods, BEAMing only allows the screening of known mutations, furthermore it also has a complex workflow and a high cost per sample, making implementation in routine clinical settings less feasible.

### 4.4. Next-Generation Sequencing (NGS)-Based Approaches

NGS is based on the analysis of millions of short sequences from DNA molecules and their comparison to a reference sequence. Multiple applications have been developed and currently used in oncology, such as targeted sequencing and whole-exome or whole-genome sequencing. Currently, NGS demonstrates a high sensitivity and specificity; nevertheless, random error rate of sequencing platforms is between 0.1% and 1% depending on the platform used [69], making impossible the detection of rare mutations. According to this observation, protocols have been specifically improved and expanded to detect rare mutations in plasma samples. Despite its great advantage to detect multiple somatic alterations simultaneously, NGS remains an expensive and time-consuming technique. Furthermore, extensive data analysis requires highly experienced bioinformaticians to identify with high confidence relevant mutations. Nevertheless, global approaches provide more accurate mutational spectrum of the tumor than targeted analyses and may also allow detection of copy number alterations and large rearrangements [46,49,50,70].

#### 4.4.1. Deep-Sequencing Using Classical NGS Protocols

Since classic NGS experimental protocols are not fully adapted to detect rare mutations, first intents to avoid this problem have been to sequence targeted regions with deep-coverage (>10,000×) [43,71,72]. Another approach was to improve alteration detections using adapted statistical methods. Thus, determination of the base position-error rate (BPER) from control samples allowed detection of true mutations as low as 0.003% and 0.001% for indels after statistical computational [44].

#### 4.4.2. TAm-Seq

Tagged-amplicon deep sequencing (TAm-Seq) has been the first sequencing method adapted to detect rare diagnosis mutations in cfDNA [45]. It is a two-step amplification process that uses the Access Array microfluidic system from Fluidigm. A first preamplification step where all primer sets are used to capture the starting molecules present in the template is processed and is then followed by a second amplification step with limited couple of primers in the microchambers of the Access Array. This process, that is only adapted to point mutation and indels, allows the identification of cancer mutations at allele frequencies as low as 2%, with more than 97% sensitivity and specificity [45].

#### 4.4.3. Cancer Personalized Profiling by Deep Sequencing (CAPP-Seq)

More recently, a capture-based NGS ctDNA detection method, the Cancer Personalized Profiling by deep Sequencing (CAPP-Seq), has been developed [46,73]. The crucial step of this protocol is the design of biotinylated “selectors” that are complementary of previously defined recurrent mutated regions. After hybrization of the “selectors” on the regions of interest and purification, amplification is carried on the reduced library [46]. Diverse classes of mutations present in somatic samples, including single nucleotide variants, indels, rearrangements, and copy number alterations, may thus be detected depending on the designed “selectors”.

In lung cancer, this method could identify mutations in 95% of NSCLC patients with 96% specificity for mutant allele fractions down to approximately 0.02% of tumors [46]. It also has been used to detect resistant mechanism in NSCLC-roceletinib-treated patients such as *EGFR* L798I and *EGFR* C797S mutations [74]. However, CAPP-Seq is still expensive for routine laboratories, with an estimated cost of 200–300 USD [73].

#### 4.4.4. Safe-SeqS

The Safe-Sequencing System (Safe-SeqS) has been proposed as a new tool to increase the sensitivity of massively parallel sequencing system instruments for rare variants identification. A unique identifier (UID) is assigned to each template DNA molecule. Tagged template molecules are then amplified to create UID families and sequences. Variants are considered real if ≥95% of the PCR fragments with the same UID contain an identical mutation [75]. The advantage of this approach is to limit base misincorporation errors during sequencing steps or basecalling errors, and to allow rare mutation detection on commercially available sequencers. To our knowledge, Safe-SeqS has been applied to plasma samples of metatastic colorectal cancer [76] and to GIST patients [77], but not to lung cancer patients.

#### 4.4.5. Circulating Single Molecule Amplification and Re-Sequencing Technology (cSMART)

cSMART is another strategy based on a similar approach that can also reduce errors occurring during library preparation or the sequencing phase. Briefly, unique barcodes are added to the end of DNA molecules, they are then circularized by ligation with an oligonucleotide containing a 4 bp degenerate sequence, and are finally amplified using two pairs of reverse PCR targeting primers strategically designed on each side of the hotspot mutation. For detection and quantitation of the targeted mutations, unique single allelic molecules are counted and mutation levels are defined [47]. This method has been used to detect clinically *EGFR* mutations in plasma samples from patients with advanced NSCLC [48]. Despite the critical step of inverse PCR primers design, one advantage of this approach is that gene fusion with unknown partner fusion can be identified.

#### 4.4.6. Digital Sequencing

In digital sequencing experiments, each strand of a double-stranded cfDNA molecule is individually tagged, allowing custom software to compare the two complementary strands and minimize errors occurring during library preparation or the sequencing phase. The digital sequence libraries are amplified and enriched for target genes using capture probes [49]. This process allows detection of SNV, indels, copy number variations (CNVs) and fusion from ctDNA samples [49,70]. As previously described, the authors considered the per-base noise in their bioinformatical process to improve detection of true variant cells.

#### 4.4.7. Bias-Corrected Targeted Next-Generation Sequencing

Recently, Paweletz et al. [50] developed a new method for library preparations that allows the minimizing of the off-target and artifacts. Briefly, multifunctional adaptors that include sequences for single-primer amplification, barcodes for sample identification and tags for sequence identification are used during the tagging step. Small targeting probes (~40 bp) designed to be adjacent to the region of interest are used to capture the targeted regions. Each probe possesses an additional tail sequence that is complementary to a biotinylated pull-down oligonucleotide. After primer extension, captured fragments are amplified with tailed PCR primers and further sequenced. This process has been successfully used for the detection of SNV, CNV and de novo rearrangement detection in 48 ctDNA samples.

#### 4.4.8. Untargeted Sequencing

Whole exome sequencing (WES) and whole genome sequencing (WGS) allow not only the screening of mutations, but also of rearrangements and of copy number variations, providing a more global genomic profiling of ctDNA aberrations. WES method on cancer patients’ plasma has been demonstrated in several studies using hybridization-based exome enrichment technologies [78,79,80,81]. Regarding WGS sequencing, Leary and colleagues was the first group to establish genome-wide profiling from plasma samples in completion of the personalized analysis of rearranged ends (PARE) method to allow a better identification of rearranged breakpoints from ctDNA [82]. Another group ran WGS in combination with bisulfite DNA sequencing to simultaneously detect genome-wide hypomethylation and copy number aberrations from hepatocellular carcinoma plasma samples [83,84]. These large-scale methods are clinically relevant as they allowed detection of alterations in an unbiased manner; however, they are still very expensive to be carried out for clinical diagnosis.

### 4.5. Other Technologies

Recently, alternative technologies using in particular Raman spectrometer or mass-spectrometry have also been development to detect low mutations from ctDNA. Surface-Enhanced Raman Spectroscopy (SERS) nanotags is based on the generation of amplicons by conventional multiplex-PCR with a barcode at the 5′-end that enables the mutation-dependent specific hybridization of SERS-nanotags and a biotin molecule at the 3′-end that allows the specific enrichment of mutated-amplicons. Following a laser excitation, each SERS-nanotag emits a specific signal that enables an easy and direct detection of multiple mutations at the same time using a Raman spectrometer [51].

UltraSEEK (high-throughput, multiplexed, ultrasensitive mutation detection) is a Mass-spectrometry-based technology that has been designed to address the limited multiplexing ability of conventional PCR approaches [52]. Briefly, a multiplex-PCR is first performed to amplify several genes, biotinylated probes, specific of the targeted mutations, are then used to generate biotinylated-mutated-strand. Matrix-Assisted Laser Desorption/Ionization Time-of-Flight Mass Spectrometry (MALDI-TOF MS) is finally used to detect presence of mutations [52].

Of note, three other approaches have previously been described to detect *EGFR* mutations in plasma DNA from lung cancer patients, including an enzymatic-based technique [85], mass spectrometry genotyping assay [86] and denaturing high performance liquid chromatography [87]. However, these techniques have not been extensively used in routine laboratories.

## 5. ctDNA and Lung Clinical Applications

### 5.1. ctDNA at Diagnosis

Since lung cancer is often diagnosed at an advanced stage of the disease, quantification of cfDNA as an early diagnostic tool for lung cancer aroused great interest. Different studies demonstrated that concentration of cfDNA is higher in advanced grade adenocarcinoma patients than in healthy volunteers [88,89,90,91,92,93]. However, as ctDNA are not detectable in all patients with NSCLC [46,94], the use of cfDNA quantification method is currently limited for lung cancer diagnosis.

One of the most important potential applications for ctDNA in lung cancer diagnosis is the detection of genetic alterations when tumor tissue is not accessible or tissue biopsy DNA extracted is not amplifiable. Many studies have demonstrated whether genetic variations within ctDNA reflects the tumor tissue mutational landscape (Table 2). Interestingly, although specificity is near 100% regardless of the technique used, the sensitivity is usually weaker (Table 2) and may depend on the alterations’ type. Indeed, *EGFR* T790M mutation showed a significantly lower detection in plasma compared to other *EGFR* alterations [67,68,95]. However, *EGFR* mutational detection in ctDNA remains a relevant alternative when the diagnostic tissue biopsy is not available [96].

### 5.2. ctDNA as a Prognostic Marker

ctDNA in lung cancer patients as the new prognostic and predictive tool has been extensively studied and challenged. Indeed, several studies report that high levels of cfDNA result in shorter overall survival (OS) [108,109], whereas other reports show that increased levels of cfDNA are not associated with OS or progression-free survival (PFS) [88,110]. These contrasting results indicate that cfDNA quantification has a limited prognostic value that can result, to some extent, from differences of plasma processing protocols used in the different studies. In contrast, quantification of *EGFR* mutations in cfDNA seems to be more relevant. Patients with high circulating *EGFR* copy number levels have a lower OS and PFS than patients with low *EGFR* copy number levels in plasma [111]. Furthermore, patients with high levels of *EGFR* activating mutations in TKI-naive plasma sample.have longer OS and PFS [103,111]. Regarding the prognostic value of *KRAS* mutation levels in the plasma of lung cancer patients, discordances are reported and were recently reviewed by Garzón and colleagues [112]. Whereas some studies show that patients with detectable *KRAS* mutation have a significantly shorter OS and/or PFS compared to wild-type patients [101,113], no differences between the two groups are reported in a recent study and a meta-analysis [14,114]. Altogether, even if some evidence suggests that *EGFR* status seems to be a more informative prognostic tool than *KRAS* in plasma samples, a reliable cut-off still needs to be determined.

### 5.3. ctDNA and Lung Cancer Tumor Burden

Another clinical application of cfDNA levels is that they may reflect the total body disease burden and surpass medical imaging for cancer detection. High cfDNA levels are significantly associated with the number of metastatic sites and tumor volume at diagnosis [46,115]. Despite that, Nygaard and colleagues found no correlation between cfDNA levels and tumoral volumetric parameters assessed by positron emission tomography (PET) scans [108], suggesting that cfDNA do not mirror a simple measurement of tumor burden. These discrepancies may primarily be attributed to differences in the methods employed for extraction and quantification.

Currently, PET scans allow the routine radiologic evaluation of treatment response to early detected signs of local recurrences or metastases. However, medical imaging is not always easily accessible, patients are exposed to ionizing radiations, and the metastases need to have reached a significant volume to be detectable. Sozzi et al. [88] first reported a link between an increase in cfDNA levels and further development of metastases or recurrence in the patients. More recently, *EGFR* mutation from plasma samples has successfully been assessed for early evaluation of the TKI treatment efficiency corresponding to the early radiologic response evaluated by chest X-rays [116]. Newman et al. [46] reported similar correlation between ctDNA levels and treatment-related imaging changes. Altogether, these studies emphasize the powerful potential of ctDNA in the follow-up of lung cancer patients in order to evaluate earlier relapse or to identify patients with residual disease. Recently, Thompson et al. [70] demonstrated the feasibility of multiple ctDNA mutation detections for lung cancer patient management using NGS.

### 5.4. ctDNA in Treatment Efficiency Monitoring

ctDNA also offers the possibility to detect acquired resistance mechanisms, including the second T790M mutation of *EGFR*, amplification of *MET* or *HER2*, and mutations of *PIK3CA* or *BRAF*, for early stage lung cancer patients under first-generation TKI medication [70]. Taniguchi et al. [41] identified the T790M mutation in ctDNA in 43.5% (10/23) of patients who had progressive disease after *EGFR*-TKI treatment. Another study also proved that sequencing of plasma DNA could complement current invasive approaches to identify mutations associated with acquired drug resistance in advanced cancers [79]. In this study, *EGFR* T790M mutation could be detected in plasma during the progression, but not at the initiation of treatment for NSCLC gefitinib-treated patients. More recently, the monitoring of T790M apparition in the ctDNA of the first-generation *EGFR*-TKI treated patients showed an average period of 2.2 months before clinical disease progression [39]. Recently, Oxnard et al. [67] proposed that the T790M ctDNA genotyping should warrant the relevant monitoring of patients treated by osimertinib (AZD9291) prior to undergoing a tumor biopsy.

The specific *EGFR* C797S mutation was also successfully detected in ctDNA in patients who developed resistance to osimertinib [117]. Interestingly, patients who relapsed under rociletinib, a third-generation *EGFR*-TKI, harbored other mechanisms of resistance including increase of *MET* copy number and *EGFR* L798I mutation [74].

## 6. Conclusions and Perspectives

Although analysis and detection of ctDNA have been asserted many years ago, liquid biopsy has recently emerged as a new potential attractive blood-based biomarker with multiple clinical applications for lung cancer patients including primary molecular diagnosis of tumors, resistant mechanisms monitoring to adapt treatments, and cancer prediction outcomes.

The relatively low sensitivity observed in the different studies reported to date can probably be explained by the lack of consensus in the choice of technical approaches, preferred sample type (serum vs. plasma), storage conditions, detected candidate mutation or suitable detection techniques (Table 2). Therefore, in order to complete the analytic and clinical validations of the sensitivity, specificity and accuracy of such liquid biopsy tests and provide the standardization of all experimental steps, ctDNA-based large-scale studies including internal validation (training and test sets) and external validation should be proposed. Targeted approaches could be taken as references since they certainly have a higher analytic sensitivity than untargeted approaches [118,119].

The use of ctDNA within the scope of clinical trials shows significant benefits and will certainly be more considered in the next years. Ultimately and more specifically the patient will highly benefits from the incorporation of this technology into the standard of care. Whether ctDNA provides a complementary or even an adequate alternative to the gold standard tumor biopsies in the near future remains the subject of many speculations. Current limitations that have been reported in many studies such as reduced sensitivity to detect some mutations in ctDNA compared to tissue biopsies should no longer remain an issue, especially because of the constant improvement in genomic approaches.

## Figures and Tables

**Table 1:** Features of techniques used to detect alterations from circulating tumoral DNA (ctDNA).

Techniques	Limit of Detection	Number of Targets	Type of Alteration Detection	Reference
PCR-based approaches				
COLD-PCR	0.10%	1	SNV, indels	[34]
PNA-LNA	0.10%	1	SNV, indels	[35]
Probes improvement	0.01%–0.10%	1	SNV, indels	[36,37]
Digital PCR	0.01%–0.10%	1 to 4	SNV, indels, CNV	[38,39,40]
BEAMing	0.01%	1 to 20	SNV, indels	[41,42]
NGS-based approaches				
Deep sequencing	0.02%	Panel	SNV, indels	[43]
Base position-error rate correction	0.003%	Panel	SNV, indels	[44]
TAm-Seq	2.00%	Panel	SNV, indels	[45]
CAPP-Seq	0.02%	Panel	SNV, indels, CNV, rearrangements	[46]
cSMART	0.01%	Panel	SNV, indels, rearrangements	[47,48]
Digital sequencing	0.10%	Panel	SNV, indels, CNV, rearrangements	[49]
Bias-Corrected Targeted NGS	0.10%	Panel	SNV, indels, CNV, rearrangements	[50]
SERS-nanotags	0.10%	1 to 3	SNV	[51]
UltraSEEK	0.10%	1 to 7	SNV, indels	[52]

PCR, polymerase chain reaction; COLD-PCR, coamplification at lower denaturation temperature PCR; PNA-LNA, peptide nuclei acid-locked nucleic acid; BEAMing, beads, emulsion, amplification, and magnetics; NGS, next-generation sequencing; TAm-Seq, tagged-amplicon deep sequencing; CAPP-Seq, cancer personalized profiling by deep Sequencing; SERS, surface-enhanced raman spectroscopy; UltraSEEK, high-throughput, multiplexed, ultrasensitive mutation detection; SNV, single nucleotide variation; CNV, copy number variation.

**Table 2:** Concordance of alteration detections in ctDNA and tissue specimen in lung cancer.

Targeted Genes	Technical Approach		Number of Plasma Samples	Performance			Reference
	Principle	Method		Sensitivity (%)	Specificity (%)	Concordance (%)	
*KRAS*	PCR-based	COLD-PCR	82	95.7	94.9	95.1	[53]
*EGFR*	PCR-based	PNA-LNA	30	79.2	100	~80	[56]
*EGFR*	PCR-based	Therascreen	652	65.7	99.8	94.3	[96]
*EGFR*	PCR-based	PNA-adapted method	97	78.3	100	ND	[97]
*EGFR*	PCR-based	Cobas	32	50	69.2	60	[98]
*EGFR*	PCR-based	Cobas	238	75	96	88	[99]
*EGFR*	PCR-based	Cobas	110				[95]
del19/L858R				73.3	100	79.8	
T790M				63.6	98.4	82.8	
*EGFR*	PCR-based	Cobas	38				[68]
del19				86	100	89	
L858R				90	100	97	
T790M				41	100	57	
*EGFR*	PCR-based	Therascreen	38				[68]
del19				82	100	87	
L858R				78	100	95	
T790M				29	100	48	
*EGFR*	PCR-based	PCR-restriction fragment length polymorphism	111	35.6	95.5	71	[100]
*KRAS*	PCR-based	PCR-restriction fragment length polymorphism	120	77	95	93	[101]
*EML4-ALK* rearrangement	PCR-based	Taqman probes	32	21	100	66	[102]
*KRAS*	dPCR	Droplet-based	64	78	100	-	[14]
*EGFR*	dPCR	Droplet-based	73	-	-	74	[103]
*EGFR*	dPCR	Droplet-based	46	66.7	100	84.8	[104]
*EGFR*	dPCR	Droplet-based	38				[68]
L858R				90	100	97	
T790M				71	83	74	
*EGFR*	dPCR	Microfluidic-chamber-based	35	92	100	-	[105]
*EGFR*	BEAMing	BEAMing	44	72.7	-	73	[41]
*EGFR*	BEAMing	BEAMing	216				[67]
del19				82.3	97.5	-	
L858R				86.3	96.5	-	
T790M				70.3	69	-	
*EGFR*	BEAMing	BEAMing	38				[68]
del19				93	100	95	
L858R				100	93	95	
T790M				71	67	70	
*EGFR*, *KRAS*, *BRAF*	NGS-based	Deep sequencing	21	100	100	100	[43]
*EGFR*, *KRAS*, *BRAF*, *ERBB2*, *PIK3CA*	NGS-based	Deep sequencing	68	58	87	68	[71]
*EGFR*	NGS-based	Deep sequencing	288				[72]
del19				50.9	98	-	
L858R				51.9	94.1	-	
*EGFR*	NGS-based	Digital sequencing	50	-	-	97.5	[70]
Panel	NGS-based	Digital sequencing	165	85	99.6	99.3	[49]
*EGFR*	NGS-based	CAPP-Seq	43	95	100	91	[74]
*EGFR*, fusion	NGS-based	CAPP-Seq	13	85	96	-	[46]
*EGFR*	NGS-based	cSMART	61	71.8	70	90.5	[48]
*KRAS*, *EGFR*	NGS-based	Capture	31	-	-	71	[94]
*EGFR*, *KRAS*, *PIK3CA*, fusion	NGS-based	Capture	39	68.5	100	78.2	[106]
*EGFR*, fusion, CNV	NGS-based	Bias-corrected	48	77	100	86	[50]
*EGFR*	Mass spectrometry	MALDI-TOF	31	80	52.4	61	[86]
*EGFR*	DHPLC		230	81.8	89.5	87	[87]
*EGFR*	Meta-analysis		3110	63	95.9	-	[107]

PCR, polymerase chain reaction; COLD-PCR, coamplification at lower denaturation temperature PCR; PNA-LNA, peptide nuclei acid-locked nucleic acid; ND, not done; dPCR, digital PCR; BEAMing, beads, emulsion, amplification, and magnetics; NGS, next-generation sequencing; CAPP-Seq, cancer personalized profiling by deep Sequencing; cSMART, circulating single molecule amplification and re-sequencing technology; MALDI-TOF, matrix-assisted laser desorption/ionization-time of flight; DHPLC, Denaturing high performance liquid chromatography; CNV, copy number variation.
